# Automated measurement of long-term bower behaviors in Lake Malawi cichlids using depth sensing and action recognition

**DOI:** 10.1038/s41598-020-77549-2

**Published:** 2020-11-25

**Authors:** Zachary V. Johnson, Manu Tej Sharma Arrojwala, Vineeth Aljapur, Tyrone Lee, Tucker J. Lancaster, Mark C. Lowder, Karen Gu, Joseph I. Stockert, Rachel L. Lecesne, Jean M. Moorman, Jeffrey T. Streelman, Patrick T. McGrath

**Affiliations:** 1 School of Biological Sciences, Georgia Institute of Technology, Atlanta, GA 30332 USA; 2 Interdisciplinary Graduate Program in Quantitative Biosciences, Georgia Institute of Technology, Atlanta, GA 30332 USA; 3 Parker H. Petit Institute of Bioengineering and Bioscience, Georgia Institute of Technology, Atlanta, GA 30332 USA; 4 Department of Computer Science, Georgia Institute of Technology, Atlanta, GA 30332 USA; 5 School of Physics, Georgia Institute of Technology, Atlanta, GA 30332 USA

**Keywords:** Social evolution, Data acquisition, Image processing, Social behaviour

## Abstract

In the wild, behaviors are often expressed over long time periods in complex and dynamic environments, and many behaviors include direct interaction with the environment itself. However, measuring behavior in naturalistic settings is difficult, and this has limited progress in understanding the mechanisms underlying many naturally evolved behaviors that are critical for survival and reproduction. Here we describe an automated system for measuring long-term bower construction behaviors in Lake Malawi cichlid fishes, in which males use their mouths to sculpt sand into large species-specific structures for courtship and mating. We integrate two orthogonal methods, depth sensing and action recognition, to simultaneously track the developing bower structure and the thousands of individual sand manipulation behaviors performed throughout construction. By registering these two data streams, we show that behaviors can be topographically mapped onto a dynamic 3D sand surface through time. The system runs reliably in multiple species, across many aquariums simultaneously, and for up to weeks at a time. Using this system, we show strong differences in construction behavior and bower form that reflect species differences in nature, and we gain new insights into spatial, temporal, social dimensions of bower construction, feeding, and quivering behaviors. Taken together, our work highlights how low-cost tools can automatically quantify behavior in naturalistic and social environments over long timescales in the lab.

## Introduction

Animals have evolved diverse behaviors that are critical for survival and reproduction, and understanding their mechanistic bases is a fundamental goal of biology. It is useful to describe and analyze different behaviors at different timescales. For example, analyses at sub-second timescales are necessary for understanding many behaviors like escape responses to predators in *Drosophila* flies^1^ and movements of individual fish swimming in large schools^2^. In contrast, analyses across many hours or days are needed to understand other behaviors including migration^2–4^, foraging^5–7^, social behaviors^8–11^, and construction behaviors^12,13^. Despite their pervasiveness in nature, we know little about the biology of these “long-term” behaviors due to several inherent difficulties of studying them in controlled laboratory settings. Understanding the mechanisms that coordinate long-term behaviors will require tools for measuring behavior over extended time periods in complex, naturalistic, dynamic, and often social environments.

Many laboratory behavioral paradigms and behavioral analysis tools are designed for individual test animals behaving alone in simple, static, unfamiliar, and unnatural environments over short timescales. In contrast, many naturally evolved behaviors are expressed in complex and changing environments, over long timescales, and through direct interaction with other individuals and/or with the environment itself. However, analyzing behaviors over long timescales in naturalistic and social conditions is challenging. For example, individuals are difficult to track in complex and changing environments, and through occlusions caused by other individuals. Furthermore, investigation over long timescales requires collection, storage, and analysis of large volumes of data. Because of these and other challenges, natural behaviors are often investigated in highly unnatural laboratory paradigms, measured only partially (e.g. for only a portion of the behavior, or through analysis of brief, intervaled observation periods), and often require manual annotation by trained experimenters. Automated approaches that circumvent these challenges will thus facilitate investigation of the biological mechanisms underlying natural behaviors.

In this paper we use low-cost automated tools to measure long-term bower construction behaviors in naturalistic social environments, for up to weeks at a time, and in multiple Lake Malawi cichlid species. Lake Malawi is the most species-rich freshwater lake on Earth, home to an estimated 700–1,000 cichlid species that have rapidly evolved in the past 1–2 million years^14^. These species vary strongly in many complex traits, including behavior^15–18^. About 200 Lake Malawi species exhibit long-term social bower construction behaviors, in which males construct large courtship structures, or bowers, during mating contexts^19^. Bower behaviors appear to be an example of convergent mating system evolution, mirroring that of Ptilonorhynchidae birds, in which males congregate into leks and construct elaborate bowers for courtship and mating, but not for raising offspring^20^. Among bower species in Lake Malawi, “pit-digging,” or excavation of crater-like depressions, and “castle-building,” or construction of volcano-like elevations, are two major variations of bower construction that have repeatedly evolved^19^. Both pits and castles are constructed over the course of many days by collecting mouthfuls of sand and spitting the sand into new locations, ultimately giving rise to the final bower structures.

Bower construction is an excellent opportunity to understand natural variation in long-term goal-directed behavior. However, bower construction is difficult to study in the lab. Bowers are often constructed for multiple hours a day over the course of many days, and thus a complete quantitative description of the behavior requires collection and analysis of large volumes of data. Bowers are constructed in social environments in which the subject male and several females can freely interact, and thus individual tracking is difficult. Bowers are constructed through sand manipulation, which continuously changes the physical environment; and the subject male and stimulus females are largely camouflaged against the sand from a top-down view, both posing difficulties for traditional computer vision strategies. Lastly, in addition to feeding on zooplankton in the water column, many sand-dwelling cichlids sift through sand for food by collecting sand into their mouths, churning it, and then either spitting it back out or filtering it through their opercula^21^. Thus, sand manipulation during bower construction is behaviorally similar to sand manipulation during feeding. Because feeding behavior is frequently performed by both male and female fish, this greatly increases the difficulty of selectively measuring bower construction behaviors from video data.

Here we describe a low-cost and automated system for measuring bower construction in laboratory aquariums. We integrate two orthogonal methods, depth sensing and action recognition, to automatically track both the developing bower structure and thousands of construction, feeding, and courtship behaviors over the course of many days, in multiple species, and in many aquariums simultaneously. The core hardware components include a mini-computer, a video game depth sensor, and a high-definition mini camera. We show that this system captures species differences in bower structure and construction behavior that mirror species differences in Lake Malawi. By registering these two data streams, we show that construction behaviors can be linked to the physical bower structure, enabling analysis of behavioral trajectories in environments that are undergoing continuous measurable change. Using this system, we analyze temporal, spatial, and social dimensions of bower construction, feeding, and quivering (a courtship behavior in which males rapidly vibrate and display their anal fins for reproductive females), that would be difficult and impractical to quantify through manual annotation. We show that (i) bower construction unfolds through punctuated bursts of activity, (ii) bower construction is performed at different times and in different locations relative to feeding behaviors, (iii) bower construction is performed at different times but in the same locations as quivering behaviors, and (iv) bower construction, feeding, and quivering behaviors are expressed in different social contexts. Taken together, our work shows how low-cost technologies can facilitate large-scale and controlled laboratory investigations of behavior over extended time periods in naturalistic environments.

## Results

### Assay and recording system for measuring bower construction behaviors

**Figure 1:**
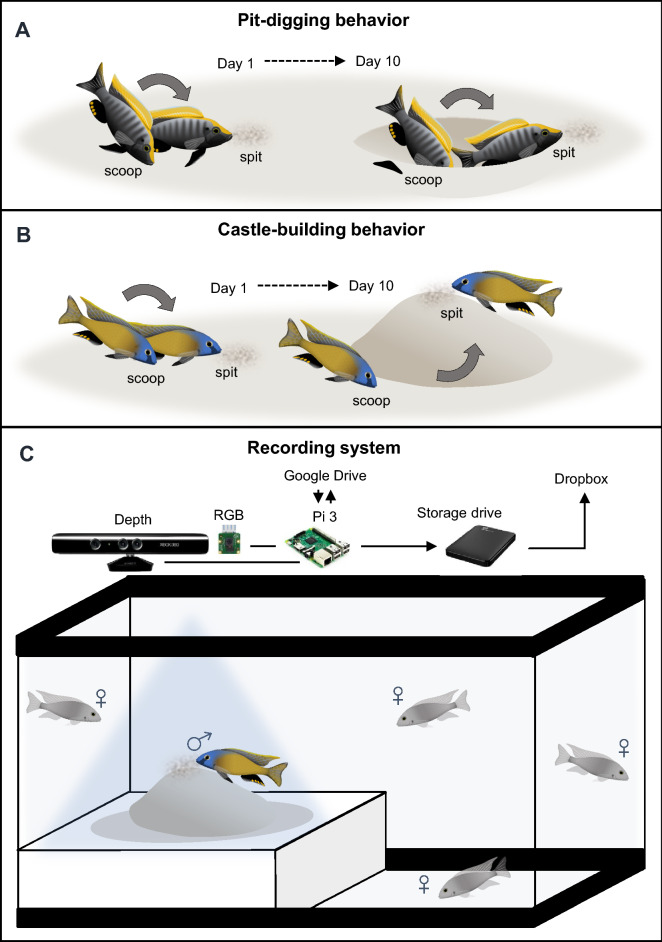
An automated recording system to measure bower behaviors in laboratory aquariums. Bowers are constructed over the course of many days (**A**,**B**). Pit-digging involves scooping sand from a concentrated region and spitting it into dispersed locations ((**A**), representation of a *Copidachromis virginalis* male digging a pit). Castle-building involves scooping sand from dispersed locations and spitting it into a concentrated region ((**B**), representation of a *Mchenga conophoros* male building a castle). To measure bower behaviors, we developed a behavioral assay and an automated recording system for standard laboratory aquatics facilities (**C**). A reproductive adult male is introduced to a 50-gallon aquarium tank containing a sand tray and four reproductive females. The recording system utilizes a Raspberry Pi 3 computer connected to a high-definition RGB camera and a Microsoft Kinect depth sensor for video recording and depth sensing, respectively. Data is stored on an external hard drive and uploaded to Dropbox. The system is remotely controlled by custom Python scripts and a Google documents spreadsheet.

Lake Malawi bower-building cichlids construct species-typical bowers in aquariums similar to those observed in the field^22^. However, because bowers are constructed intermittently over the course of many days, we found that daily 2–3 h video recordings were insufficient for capturing behavior consistently. To capture construction behavior more consistently, we designed a system capable of recording 10 h of video data and 24 h of depth sensing data daily for 10 days. Briefly, we mounted small, inexpensive Raspberry Pi 3 (Pi) computers above each aquarium, and each unit was connected to a small touch screen, an external hard drive for data storage, and an ethernet cord for internet access and interfacing with a common Google spreadsheet file (Figs. 1, S1–S3). For video recording, we connected each unit to a Raspberry Pi camera board that supports HD quality compressed video with a high frame rate (30 frames per second); and for depth sensing, we connected each unit to a Microsoft Kinect depth sensor, which has previously been shown to capture structural features of creek beds through shallow water^23^. For each bower trial, a subject male was introduced to a 50-gallon aquarium containing four adult reproductive females and a sand tray positioned directly beneath the Raspberry Pi camera and Kinect depth sensor for top-down recording (Fig. 1C, also see Figs. S1, S3).

### Depth data

#### System validation: the Kinect detects surface change during bower construction

**Figure 2:**
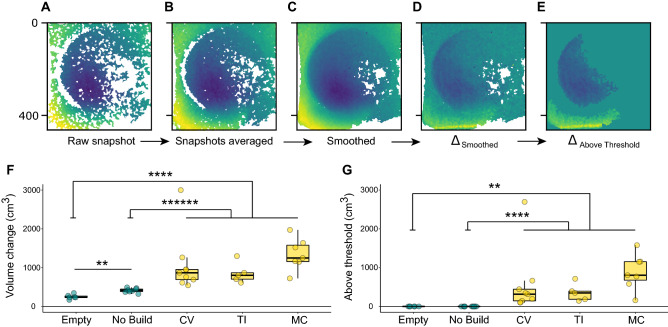
Depth sensing detects structural change associated with bower construction. The Kinect collects top-down depth snapshots of the sand tray surface through time (**A**–**E**), with yellow indicating distances closer to Kinect (elevated regions), and dark blue indicating regions farther from the Kinect (depressed regions). Raw Kinect depth snapshots of the sand tray surface contained ~ 40% missing data (white pixels; (**A**)). To improve depth data quality, consecutive depth snapshots were collected and averaged together every 5 min, reducing the proportion of missing data to ~ 20% (**B**). Data quality was further improved by spatially interpolating data in small NaN “islands,” reducing the proportion of NaN pixels to ~ 10% (**C**). Depth change over the course of the trial was calculated by subtracting the initial depth map from the final depth map, with turquoise indicating no change (**D**). Thresholding enabled depth change signals caused by bower construction to be separated from signals caused by noise and other home tank activities (**E**). Before thresholding, total volume change differed strongly between control trials (Empty tank trials, and trials in which no bower was constructed) and bower construction trials (**F**). Following thresholding, all bower trials exhibited above threshold volume change while control trials did not (**G**).

To validate measurements of depth change, we analyzed the total volume of sand moved in “bower” trials (in which an experimenter visually identified bowers constructed by the male; n = 29 total; pit-digger *Copadichromis virginalis,* CV, n = 9; castle-builder *Mchenga conophoros*, MC, n = 7; pit-digger *Tramitichromis intermedius,* TI, n = 5) and control trials in which males and females fed in the sand but no bowers were constructed (n = 9 total; CV, n = 3; MC, n = 3; TI, n = 3) by subtracting the initial depth map from the final depth map (for visualization of this calculation see Fig. 2A–E). As a second control, we also analyzed empty tank (no fish) trials to estimate the level of depth change that might be attributed to technical noise. Because males move large volumes of sand during bower construction, we expected to observe larger depth change signals in bower trials compared to control trials. We found that depth change differed strongly between these three conditions (Kruskal–Wallis χ^2^ = 27.2, p = 1.22 × 10^–6^), and was much greater in bower construction trials (n = 21 trials; 1111.8 ± 124.68 cm^3^ volume change) compared to control trials (n = 9 trials; 414.5 ± 17.53 cm^3^ volume change; Wilcoxon Rank-Sum Test, Benjamini–Hochberg adjusted p = 4.19 × 10^–7^) and compared to empty tank trials (n = 6 trials; 249.9 ± 24.00 cm^3^ volume change; Wilcoxon Rank-Sum Test, Benjamini–Hochberg adjusted p = 1.01 × 10^–5^, Fig. 2F,G).

#### Biological validation: depth sensing captures natural species differences in bower structures

**Figure 3:**
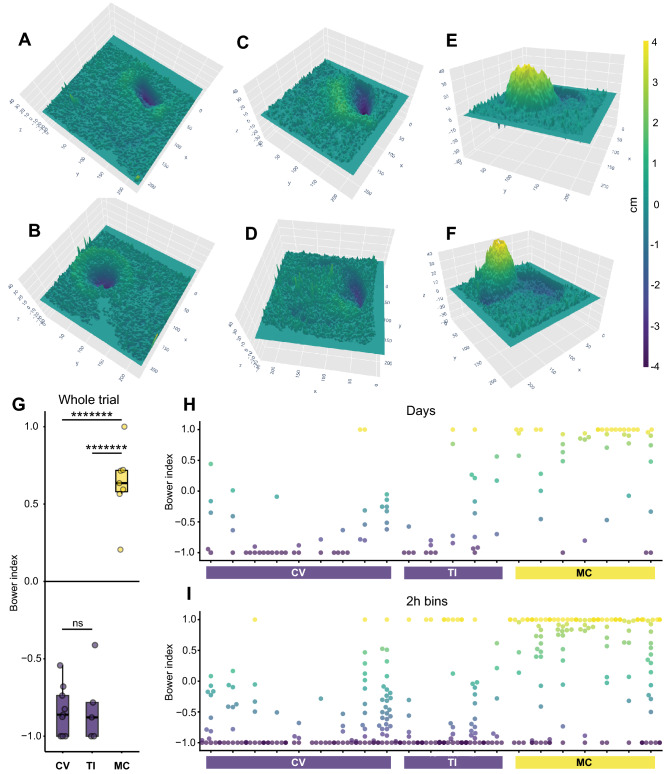
Depth sensing reveals natural species differences in bower construction at multiple timescales. Representative 3D reconstructions show pits constructed by *Copadichromis virginalis* (CV, n = 9; (**A**,**B**)), pits constructed by *Tramitichromis intermedius* (TI, n = 5; (**C**,**D**)), and castles by *Mchenga conophoros* (MC, n = 7; (**E**,**F**)); all z-axes amplified for visual effect). The Bower Index, a measure of the directional bias in bower construction, revealed strong species differences in final bower structure (whole trial change, e.g. (**A**–**F**)). MC exhibited a positive bias (extreme depth change regions tended to be elevated) and differed strongly from both TI and CV, which exhibited negative biases and did not differ significantly from each other (**G**). Strong species differences in structural development were also present when depth data was analyzed at daily (**H**) and hourly (I, 2-h bins) timescales, mirroring the direction of differences observed for whole trial change. Each point represents the observed Bower Index for a single time bin within one trial. Different individuals are separated into columns along the x-axis, grouped by species. The color of each point reflects the Bower Index for that time bin (− 1, purple, representing pure pit-like depth change; 1, yellow, representing pure castle-like depth change).

We next tested whether our depth sensing system could detect natural species differences in pit-digging versus castle-building. To do this, we compared depth change in bower trials among three species: two pit-digging species (*Copadichromis virginalis*, n = 9; *Tramitichromis intermedius*, n = 5) and one castle-building species (*Mchenga conophoros*, n = 7). Subtraction of the initial surface depth from the final surface depth enabled quantification and 3D reconstruction of the final bower structure constructed by each male (Fig. 3A–F). We calculated a “Bower Index” to analyze the final bower structure in each trial (Fig. 3G). Briefly, the Bower Index is a ratio of the net depth change in above threshold regions (change can be positive or negative) to the total volume of depth change in above threshold regions (all change is considered positive). The Bower Index is thus a scaled measure of directional bias (elevation vs. depression) in regions that undergo above-threshold structural change. This analysis revealed strong species differences in bower structures (One-way ANOVA, p = 5.42 × 10^–11^), with all pit-digging individuals exhibiting a negative Bower Index (14/14), and all castle-building individuals exhibiting a positive Bower Index (7/7). Post-hoc Tukey’s HSD tests revealed that pit-digging species did not differ significantly from each other (CV vs. TI, Tukey’s HSD, p = 0.98; Fig. 3G), but the castle-builder *Mchenga conophoros* differed strongly from both pit-digging species (MC vs. TI, Tukey’s HSD, p = 1.68 × 10^–9^; MC vs. CV, Tukey’s HSD, p = 1.16 × 10^–10^). Strong species differences in structural development were also present when depth data was analyzed at daily (24-h bins; One-way ANOVA, p = 4.95 × 10^–8^; H) and hourly (2-h bins; One-way ANOVA, p = 1.62 × 10^–11^; I) timescales, mirroring the directional pattern of differences observed at the whole-trial level (Fig. 3H,I).

### Combined video and depth data

We previously developed a video analysis pipeline that uses a trained 3D Residual Network (3D ResNet) to automatically identify cichlid bower construction, feeding, and quivering behaviors from raw 10-h videos collected daily during behavioral trials^24^. Briefly, 3D ResNets are 3D Convolutional Neural Networks (CNNs) that incorporate features of ResNets, in which signals are bypassed across convolutional layers during training. This approach allows 3D ResNets to be deeper and more accurate than traditional 3D CNNs for action classification tasks^25^. Using the trained 3D ResNet, we annotated more than 550,000 total sand disturbance events from seven different behavioral trials.

#### System validation: registration links behavioral events to depth data through time

We spatially and temporally aligned CNN-predicted behavioral events with depth data for the seven trials (described immediately above). We used RGB images collected with the Kinect for spatial registration of video and depth data, and we used time stamps assigned by the Raspberry Pi for temporal alignment. We found that most (~ 56%) of CNN-predicted events could be linked to sand surface height at the corresponding time and location. We also found a large proportion (~ 44%) of CNN-predicted events could not be linked to surface height, which was not surprising because the video FOV included the glass walls outside the sand tray, and as much as ~ 10% of the sand surface was not captured by the Kinect in each trial. We observed a bias in the types of events that could not be linked to depth change values, with just five categories accounting for ~ 87% of these predictions (shadow/reflections: 31.9%, other: 21.3%, feed multiple: 14.2%, feed scoop: 12.7%, and feed spit: 6.4%). This bias is not surprising, as fish frequently feed along the periphery against the glass walls, and “shadow/reflections” includes reflections of events in the glass. In contrast, bower scoops and spits represented a small minority of these events (bower scoop: 3.4%, bower spit: 4.1%), supporting the idea that high quality depth data was collected in regions where the males constructed bowers.

#### Biological validation: agreement between action recognition and depth sensing

**Figure 4:**
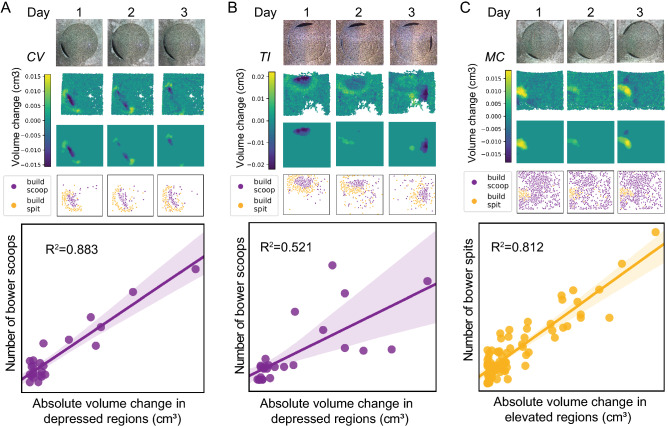
CNN-predicted construction behaviors are strongly correlated with bower structure. RGB images collected with the Kinect (first row, (**A**–**C**)) were registered to RGB frames collected with the Raspberry Pi Camera to spatially align video and depth data. Daily depth change data (second row, (**A**–**C**)) was analyzed to identify above-threshold regions (third row, (**A**–**C**)). In pit-diggers (**A**,**B**), a greater proportion of CNN-predicted bower scoops versus bower spits mapped onto extreme height change regions (overlap of third and fourth rows), whereas in castle-builders the reverse was true: a greater proportion of bower spits versus bower scoops mapped onto extreme height change regions. In pit-diggers, the number of bower scoops per hour was strongly and positively correlated with the total volume change in that hour (e.g. see representative regression plots for individual trials in (**A**,**B**), whereas in castle-builders the number of bower spits per hour was strongly and positively correlated with the total volume change in that hour (regression plot, (**C**)).

Using registered video and depth data, we tested how CNN-predicted scoop and spit events mapped onto bower structures identified from depth data (Fig. 4). Because pits are excavated by scooping sand, we predicted that a greater number of scoops compared to spits would occur within the most extreme depth change regions of interest (bower ROIs) in pit-diggers, and that the opposite pattern would be observed in castle-builders. To test this, we compared the number of scoops and spits observed inside and outside the bower ROI for each of the five parental trials analyzed by the 3D ResNet (n = 1 CV, n = 2 TI, n = 2 MC). Indeed, pit-diggers performed ~ 15x more CNN-predicted scoops versus spits within daily bower ROIs and this pattern was highly significant within each subject (CV: 273 scoops vs. 20 spits, χ^2^ = 311.35, p < 2.2 × 10^–16^; TI subject 1: 339 scoops vs. 16 spits, χ^2^ = 127.2, p < 2.2 × 10^–16^; TI subject 2: 602 scoops vs. 60 spits, χ^2^ = 377.28, p < 2.2 × 10^–16^). This pattern was reversed in castle-builders, with ~ 5.5x more CNN-predicted spits versus scoops being performed within daily bower ROIs (MC subject 1: 242 scoops vs. 2208 spits, χ^2^ = 5554.2, p < 2.2 × 10^–16^; MC subject 2: 260 scoops vs. 462 spits, χ^2^ = 208.92, p < 2.2 × 10^–16^).

We also tested if CNN-predicted bower behaviors were predictive of structural change measured by depth sensing over shorter timescales. In pit-diggers, we found that the number of hourly bower scoops was strongly and positively correlated with the hourly volume change in depressed regions (R^2^ = 0.597, p < 0.00001); whereas in castle-builders, the number of hourly bower spits was strongly and positively correlated with the hourly volume change in elevated regions (R^2^ = 0.690, p < 0.00001, representative trials shown in Fig. 4A–C). Taken together, these data show that behaviors identified through action recognition are tightly linked to the spatial, geometric, and temporal development of the bower structure measured through depth sensing, indicating agreement between these two orthogonal data streams.

### Temporal, spatial, and social dimensions of bower construction

#### Bowers are constructed through punctuated bursts of activity

**Figure 5:**
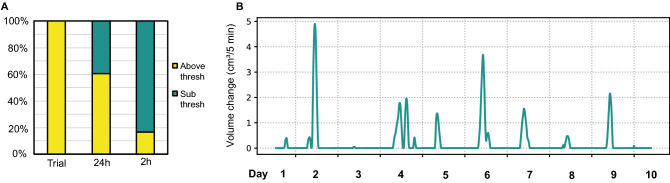
Bowers are constructed through punctuated bursts of activity. Following thresholding, all bower trials exhibited above threshold volume change. At shorter timescales, 60.6% of all 24-h bins analyzed and 16.7% of all 2-h bins analyzed contained above threshold depth change (**A**). Analysis of rolling hourly depth change in five-minute increments over the course of whole trials reveals that structural change is driven by relatively brief bursts of activity punctuated throughout trials (representative *Mchenga conophoros* trial, (**B**)).

In addition to measuring the final bower structure, we also used depth data to investigate whether bowers are constructed gradually and uniformly over time or non-uniformly in bursts. To quantify temporal activity patterns within bower trials, we first split depth data from each trial into 2-h and 24-h bins, and then analyzed the proportion of bins containing above-threshold depth change. At the daily level, we found that 60.6% of all 24-h bins analyzed contained above-threshold depth change, indicating that males constructed on the majority of days throughout the trial. In contrast, just 16.7% of 2-h bins analyzed contained above-threshold depth change (Fig. 5A). These data suggest that bowers are constructed through punctuated bursts of activity (representative *Mchenga conophoros* trial, Fig. 5B).

#### Construction, feeding, and quivering behaviors are expressed at distinct times

**Figure 6:**
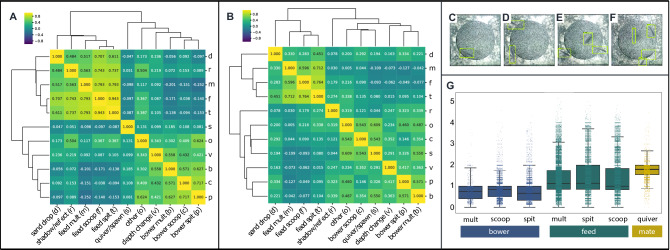
Temporal and spatial covariance among depth change, bower construction, feeding, and spawning. Hierarchical clustering of depth change and 3D ResNet-predicted behavioral events through time reveals three largely distinct behavioral clusters corresponding to feeding (left-most columns), bower construction (right-most columns), and quivering behaviors (middle column, (**A**); Pearson’s R values shown for each pairwise correlation). Quivering was more strongly and positively correlated with bower construction than with feeding. A similar analysis of the spatial distributions of depth change and behavioral events across the sand surface revealed two major clusters, one corresponding to feeding (left-most columns), and the other corresponding to quivering and construction behaviors (right-most columns; (**B**)) notably, quivering was strongly spatially correlated but weakly temporally correlated with bower construction behaviors. To analyze social context, we used object recognition to detect and count fish above the sand tray during different behaviors, with green outlines indicating predicted fish (**C**–**F**); 1, 2, 3, and 4 fish detected, respectively). Analysis of the number of fish present above the sand tray during 3D ResNet-predicted behavioral events revealed strong differences in fish count across behavioral categories ((**G**); p < 2.2 × 10^–16^). Fish counts were lowest during bower construction, greater during feeding, and greatest during spawning, supporting dynamic and interrelated behavioral and social contexts across whole trials. Pearson’s R values are shown for each pairwise comparison, and the significance of each pairwise comparison and corresponding p-values can be found in Supplementary Tables S1 and S2. Letter abbreviations for each behavioral measure are indicated in the labels along the horizontal axis.

We also wanted to understand how construction behaviors are related to other behaviors that are performed during trials, such as feeding and quivering. To do this, we first analyzed patterns of temporal covariance among different behavioral categories across whole trials. Because this analysis had the potential to reveal unexpected relationships among behaviors, we analyzed all behavioral categories as well as the volume of depth change through time. Because bower construction and quivering are both courtship behaviors, we expected them to be expressed at similar times, and therefore we expected to observe strong and positive correlations between these behaviors across bins. We also expected feeding behaviors to be expressed at different times than bower construction and quivering, and we therefore expected different feeding behaviors to be strongly and negatively correlated with bower construction and quivering. We found strong positive correlations between construction scoops and spits (Pearson’s R = 0.717, p < 2.0 × 10^–16^, Fig. 6A) and between feeding scoops and spits (Pearson’s R = 0.943, p < 2.0 × 10^–16^, Fig. 6A), but negative correlations between construction behaviors and feeding behaviors (bower scoop versus feed scoop, Pearson’s R = − 0.03802, p = 0.33; bower scoop versus feed spit, Pearson’s R = − 0.09374, p = 0.017; bower spit versus feed scoop, Pearson’s R = − 0.14039, p = 3.4 × 10^–4^; bower spit versus feed spit, Pearson’s R = − 0.15271, 9.75 × 10^–5^, Fig. 6A). Taken together, these data suggest that construction is expressed at different times than feeding. Unexpectedly, we found quivering was relatively weakly correlated with both feeding (quivering versus feed scoop, Pearson’s R = − 0.09665, p = 0.014; quivering versus feed spit, Pearson’s R = − 0.08741, p = 0.026, Fig. 6A) and construction behaviors (quivering versus bower scoops, Pearson’s R = 0.1077, p = 6.14 × 10^–3^; quivering versus bower spits, Pearson’s R = 0.080532, p = 0.041, Fig. 6A). In contrast to our predictions, these data suggest that quivering behaviors are expressed largely independently of bower construction behaviors through time. A complete list of Pearson’s R values and p-values for each pairwise relationship among behavioral categories can be found in Table S1.

#### Construction behaviors and feeding behaviors are expressed in distinct spatial locations

We also wanted to understand how different behaviors are spatially related across the sand surface. Because males typically quiver within or above the bower in the wild, we expected that quivering behaviors and bower construction behaviors would be strongly and positively spatially correlated (i.e. expressed in the same spatial locations). In contrast, we expected that feeding would be performed indiscriminately across the sand surface, and therefore that feeding behaviors would be uncorrelated with construction behaviors, quivering behaviors, and also with one another. Consistent with our expectations, bower construction behaviors and quivering behaviors were strongly and positively correlated with one another (bower scoops versus quivering, Pearson’s R = 0.5434, p < 2.0 × 10^–16^; bower spits versus quivering, Pearson’s R = 0.3262, p < 2.0 × 10^–16^; bower multiple versus quivering, Pearson’s R = 0.5501, p < 2.0 × 10^–16^, Fig. 6B); and feeding behaviors were weakly correlated with bower construction behaviors (feeding scoops versus bower scoops, Pearson’s R = 0.08970, p < 2.0 × 10^–16^; feeding scoops versus bower spits, Pearson’s R = − 0.04891, p < 2.0 × 10^–16^; feeding spits versus bower scoops, Pearson’s R = 0.1348, p < 2.0 × 10^–16^; feeding spits versus bower spits, Pearson’s R = 0.09520, p < 2.0 × 10^–16^; additional relationships among feeding and construction behaviors are shown in Fig. 6B, Table S2) and with quivering behavior (feeding scoops versus quivering, Pearson’s R = − 0.09342, p < 2.0 × 10^–16^; feeding spits versus quivering, Pearson’s R = 0.07975, p < 2.0 × 10^–16^). However, contrary to our expectations, we found strong and positive correlations among feeding behaviors (feeding scoops versus feeding spits, Pearson’s R = 0.7636, p < 2.0 × 10^–16^; feeding scoops versus feeding multiple, Pearson’s R = 0.5964, p < 2.0 × 10^–16^; feeding spits versus feeding multiple, Pearson’s R = 0.7120, p < 2.0 × 10^–16^, Fig. 6B), suggesting feeding was preferentially expressed in specific locations. Taken together, these data demonstrate that bower construction and quivering tend to be expressed in similar spatial locations, and that feeding behaviors are expressed non-randomly in locations that are largely independent from the bower region. A complete list of Pearson’s R values and p-values for each pairwise relationship among behavioral categories can be found in Table S2.

#### Construction, feeding, and quivering behaviors are expressed in different social contexts

We also aimed to understand the social contexts in which bower construction, feeding, and quivering are expressed. We integrated action recognition^24^ with object recognition to count the number of fish present above the sand tray during expression of these behaviors (see example predictions in Fig. 6C–F). Linear mix-effects regression with fish count as the outcome variable, behavior as a fixed effect, and day nested within individual nested within species as a random effect, revealed that the number of fish present above the sand tray differed strongly among behavioral categories (F = 2285.9, p < 2.2 × 10^–16^; Fig. 6G). The fewest fish were present during bower construction behaviors (average fish count regression estimates for build scoop = 0.99 ± 0.118, build spit = 0.88 ± 0.118, build multiple = 0.87 ± 0.118); a greater number tended to be present during feeding behaviors (feed scoop = 1.24 ± 0.118, feed spit = 1.27 ± 0.118, feed multiple = 1.21 ± 0.118); and the greatest number of fish, on average, were present during quivering behaviors (1.93 ± 0.118). Post-hoc pairwise comparisons revealed significant differences between all behaviors with the exception of bower spit versus bower multiple events (t = − 0.396, p = 0.997). Taken together, these data suggest that bower construction, feeding, and quivering behaviors are expressed in distinct social contexts.

## Discussion

Construction behaviors are essential for survival and reproduction in a wide variety of animal species, and are excellent natural models of long-term goal-directed behavior in a changing environment^13,20,26–28^. New tools are facilitating behavioral measurements in increasingly complex environments. For example, static poses and positions of animals can be generated from image and video data, allowing individual tracking and/or pose estimation in a variety of complex and naturalistic environments^29–31^. Depth sensing, radio-frequency identification (RFID) tagging, and multiple camera setups have been used in conjunction with standard video data to track animals in social and physical environments in which occlusions regularly occur^32–36^. However, position and pose estimation can only partially describe behaviors that are defined by interactions between animals and their environments, such as construction behaviors. In order to generate complete quantitative descriptions of these behaviors, it is necessary to measure both the physical environment and the behavior simultaneously. Achieving this will facilitate our understanding of behaviors that are widespread in nature but that remain largely unexplored in behavioral genetics and neuroscience.

In this study, our primary goal was to automatically measure both developing bower structures and the underlying construction behaviors in naturalistic social environments for extended time periods. We found that a low-cost mini-computer and depth sensor were sufficient for tracking the structural development of bowers for up to weeks at a time in multiple species and across many aquariums simultaneously. Depth sensing reliably detects signals caused by bower construction and captures natural differences between pit and castle structures that mirror species-typical structures in Lake Malawi^19^. These results highlight depth sensing as a promising tool for phenotyping many bower constructing species and hybrid crosses. Depth sensing may also be useful for measuring the development of other extended phenotype structures through time, such as underwater or above-ground nests, and for tracking activity patterns in animals that construct subterranean structures, e.g. by measuring the volume of substrate that is displaced above ground over time^37–41^.

A major strength of our system is the integration of two orthogonal data streams, depth sensing and action recognition, to simultaneously measure the structure and the underlying construction behaviors over long time periods. By registering these data streams in time and space, we show that bower construction behaviors identified through action recognition are strongly predictive of structural change measured through depth sensing. Further, we link species differences in construction behaviors to differences in the final structure: pit-digging species perform far more scoops in bower regions, and the number of scoops predicts the volume of structural change in these regions; while castle-builders perform far more spits in bower regions, and the number of spits predicts volume of structural change in these regions. Registration thus enables thousands of individual behavioral actions to be mapped onto a changing 3D surface in each behavioral trial, and will allow future studies to define the underlying behavioral logic of bower construction.

This system provided insights into temporal dimensions of bower construction, feeding, and quivering behaviors that would have been difficult or impossible to gain through manual analysis. We found that bowers develop in bursts, typically spanning half of daylight hours or less, suggesting that bowers are constructed in punctuated bouts of activity interspersed amongst extended periods of inactivity. This is consistent with field observations in which males of some species have been reported to leave their bowers for extended periods of time, presumably to feed. We also found a weak correlation between construction behaviors and quivering through time. Because bower construction and quivering are both integral to mating, we expected that these behaviors would be coordinated similarly through time, via the same reproductive hormones and external mating cues. One potential explanation for this weak correlation is that construction behaviors are triggered first, and quivering is triggered only when specific combinations of hormonal, chemosensory, and/or behavioral cues are selectively emitted by females as they approach oviposition, similar to integrations that have been demonstrated in other cichlids and mammals^42,43^. We also found a strong and negative correlation between bower construction and feeding behaviors through time, despite both behaviors occurring on the majority of days during trials. These data raise the intriguing possibility that feeding behavior may inhibit bower construction, and/or vice versa, and motivate future studies aiming to understand how feeding and construction behaviors are related.

We also gained new insights into spatial dimensions of construction, feeding, and quivering behaviors. As expected, we found strong spatial correlations between bower construction and quivering behaviors, consistent with behavior in the wild in which males quiver and spawn with females within or above the bower^21^. However, we were surprised to find a strong spatial bias in the expression of feeding behaviors: different categories of feeding events were strongly and positively correlated with one another, and were weakly or negatively correlated with bower construction and quivering behaviors. These data suggest that animals feed non-randomly across the sand surface and in largely non-overlapping spatial locations relative to the bower. One potential explanation is that feeding behaviors are preferentially expressed in specific locations regardless of the location of the bower structure, for example along edges where small particles of food debris may settle. An alternative possibility is that feeding is spatially indiscriminate prior to construction, but animals avoid the bower structure once it is present. These data further motivate future studies that aim to understand the relationships between bower construction and feeding.

To better understand the social contexts in which construction, feeding, and quivering behaviors are expressed, we integrated object detection and action recognition to count the number of fish present over the sand during these behaviors. This analysis revealed that construction, feeding, and quivering are expressed in different social contexts. We observed the fewest fish present over the sand tray during construction behaviors, consistent with males aggressively chasing away females from the bower region during construction. We found greater and highly variable fish counts during feeding behaviors. One potential explanation for this result is that male aggression is inhibited during feeding, and both the subject male and stimulus females freely and frequently enter and exit the sand tray to feed during these times. We observed the greatest number of fish present (~ 2) during quivering behaviors, and the variance around this mean was small. This was likely driven by the fact that quivering and spawning occurring exclusively between the male and a single gravid female, while other females are aggressively chased away from the bower region.

Several limitations can offer guidance for future development of this system and for other experimental paradigms. First, in this study we sacrificed temporal resolution for improved spatial resolution of depth data. Depth sensing with high temporal resolution can be used as a powerful tool for tracking animals against visually complex backgrounds, across 3D trajectories, and/or through occlusions^31,35,36,44^, and thus may be critical for the success of other paradigms. Although sacrificing temporal resolution allowed us to recover a large amount of depth data, the version of the Kinect used in this study still yielded a significant degree of data loss. Many new depth sensors with improved time-of-flight technology have been released (including the Kinect v2), but these require USB 3.0 which is not a feature of the Raspberry Pi used in this study (Raspberry Pi 3 Model B+). However, Raspberry Pi has recently released the Raspberry Pi 4, which includes USB 3.0 among other upgrades, opening the door to higher quality depth data and improved temporal resolution at relatively low cost. Another limitation is the practical challenge of remotely controlling a large set of computers and storing, transferring, and analyzing large volumes of video and depth data. For our project, this required planning with information technology professionals at our institution and a Business Dropbox Account for data storage, as well as computer science expertise for developing analysis pipelines. However, these hurdles will likely become less prohibitive as performance specifications improve on low-cost computer systems and more open source and user-friendly computational tools are made publicly available. Another limitation is that additional ethologically meaningful behaviors such as aggressive displays do not typically cause sand change in this paradigm, and therefore cannot be tracked reliably with our current pipeline. Future improvements integrating individual tracking, pose estimation, and path tracing combined with 3D ResNets trained on additional behavioral categories will enable a fuller suite of behaviors to be tracked over the course of these trials. A final limitation is that our system currently analyzes all video and most depth data after it is collected. Further improvements are needed to enable real-time processing of data, which may be necessary for some projects.

Despite these limitations, these experiments are a significant step for computational ethology, overcoming several major challenges facing the automated measurement of natural long-term behaviors in the lab. Our recording system enables automated phenotyping of naturally evolved construction behaviors in multiple wild-derived species, in naturalistic social environments, over extended time periods. Bower construction behaviors are expressed by more than 200 cichlid species spanning multiple lakes, and an even larger number of species live and feed in the sand. Our system thus lays a foundation for studying the biological basis of vertebrate behavioral evolution on large comparative scales in the lab. The system is also effective for behaviorally phenotyping interspecies hybrids, and will thus be useful for investigating the transition between two species-divergent behaviors in F_1_ hybrids^22^, and for genetic mapping of behavioral variation in F_2_ hybrids. The ability to phenotype many behaviors, and to track thousands of behavioral actions over extended time periods also makes this system promising for future neural recording experiments.

## Conclusions

We have designed, developed, and implemented a behavioral paradigm and recording system for automatically phenotyping social construction behaviors in naturalistic environments in cichlids. By integrating depth sensing and action recognition, we phenotype bower behaviors in multiple species and hybrid crosses over weeklong periods in many tanks simultaneously. This system will help accelerate comparative behavioral genetics and neuroscience experiments in one of the most powerful vertebrate systems for studying natural behavioral evolution.

## Methods

### Animals and husbandry

#### Subjects

All animals were housed and tested in accordance with the Georgia Institute of Technology Institutional Animal Care and Use Committee (IACUC) guidelines. Lake Malawi bower-building species (*Copadichromis virginalis, Tramitichromis intermedius, Mchenga conophoros*) derived from wild-caught stock populations were housed in social communities (20–30 individuals) in 50-gallon glass aquaria (90.2 cm long × 44.8 cm wide × 41.9 cm tall) into adulthood (> 180 days). Aquaria were maintained under conditions reflective of the Lake Malawi environment: pH 8.2, 26.7 °C water, and a 12 h:12 h light:dark cycle with 60-min transitional dim light periods. For all behavioral experiments, a single reproductive adult male and four reproductive adult stimulus females of the same species or hybrid background were introduced into designated home tanks (as described above) equipped with additional LED strip lighting (10 h:14 h light:dark cycle synced with full lights on), and a custom-designed hollow acrylic case (43.1 cm long × 43.1 cm wide × 10.2 cm tall, with a 35.6 cm diameter circular opening) surrounding a circular plastic tray (35.6 cm diameter × 6.4 cm deep, and elevated 3.8 cm above the aquarium bottom) filled with sand (Carib Sea; ACS00222). Sand trays were positioned approximately 58 cm directly below a Microsoft XBox Kinect depth sensor and Raspberry Pi video camera; and approximately 30 cm directly below a custom-designed transparent acrylic tank cover (38.1 cm long × 38.1 cm wide × 4.4 cm tall) that contacted the water surface to eliminate rippling for top-down depth sensing and video recordings (described below). In both stock and behavioral tanks, fish were fed twice daily with dried spirulina flakes (Pentair Aquatic Eco-Systems).

#### Behavioral trials

All animal experiments were reviewed and approved by IACUC under the protocol #A17052. For each behavioral trial, a single reproductive adult subject male was introduced to a designated behavioral tank containing four reproductive adult stimulus females and a full sand tray as described above (under “Animals and husbandry” section). Upon introduction, an automated recording protocol (described in detail below) was initiated, collecting RGB video and depth data during full light hours (08:00 to 18:00 EST) for 7–10 days. Subjects and stimulus females were allowed to freely interact throughout the entirety of the recording trial and followed the same feeding schedule described above (under “Animals and husbandry” section).

### Recording and monitoring system

#### Hardware

The automated recording system consisted of a Raspberry Pi 3 Model B (RASPBERRYPI3-MODB-1GB; Raspberry Pi Foundation) connected to the following: (1) a 7″ touchscreen display (RASPBERRYPI-DISPLAY; Raspberry Pi Foundation) secured in an adjustable mount case (Smarticase); (2) an Xbox 360 Kinect Sensor (Microsoft); (3) a Raspberry Pi camera v2 (RPI 8MP CAMERA BOARD; Raspberry Pi Foundation); and (4) a 1 TB external hard drive (WDBUZG0010BBK-WESN; Western Digital).

#### Code

We wrote custom Python scripts for all aspects of the project. All code is publicly available on github at www.github.com/ptmcgrat/Kinect2. A general outline of the code is available in the Supplementary Materials.

#### Depth sensing

We used a Microsoft Xbox Kinect depth sensor to measure the topology of the sand surface through water. The Kinect is a low-cost, close-range, high-resolution depth sensor containing an IR laser and refractor that emits a known structured light pattern, and an IR camera that detects the emitted IR light across surfaces within the FOV. The Kinect then uses a pattern recognition algorithm to compute distance of surfaces across the FOV, which can be stored into 640 × 480 numpy array files (.npy). Kinect depth sensing was controlled through a custom Python script (the CollectData function within the CichlidBowerTracker.py script, see Supplementary Materials and “Methods” section) that was initiated at the beginning of each behavioral trial. Because continuous depth data was both unnecessary and impractical (due to the large volume of high frame rate uncompressed depth data), CollectData combined depth data collected continuously at ~ 10 Hz into a single frame every 5 min. The code also specifies collection of a single RGB snapshot every 5 min, for later registration between depth data and video data. All depth data was stored on an external hard drive for later processing.

#### Video recording

The same CichlidBowerTracker.py script controlled daily collection of 10 h of 1296 × 972 RGB video through a Raspberry Pi v2 camera (Raspberry Pi Foundation) during full lights on hours (08:00–18:00 EST). The large volume of video data collected per day was enabled by instantaneous compression into .h264 format by the Raspberry Pi. Compressed video data was stored on an external hard drive for later processing.

#### Google controller spreadsheet

A Google Controller spreadsheet was created to remotely control each tank’s Raspberry Pi recording system, provide real-time visual updates of bower activity every 5 min, and logging behavioral trial information into a master datasheet. The Controller sheet served as a master graphical user interface for the recording system, with a “Command” column monitored by each Raspberry Pi. The Commands included “New” to initiate a new trial, “Restart” to resume an existing trial, “Rewrite” to overwrite an existing trial, “Stop” to stop a trial, “UploadData” to upload data from a completed trial to Dropbox, and “LocalDelete” to clear data from the local storage drive following upload. A more detailed description of Google Controller setup and functionality is provided in the Supplement (“Controller Spreadsheet”).

### Data processing and analysis pipeline

#### Data upload

Following completion of each trial, data was copied from the local external hard drive to a laboratory Dropbox account through the Google Controller spreadsheet by upload through rclone, a cloud storage sync program (https://rclone.org/). The directory for each trial contained all videos, RGB frames, and depth frames recorded for the trial. Due to the large volume of data, uploading for all data collected in a recording round (~ 10 trials, ~ 4 TB of data) typically required 24–28 h. For later trials, upload time was reduced to ~ 3–5 h by first compressing depth data into .tar files.

#### Depth analysis

Analysis of Kinect depth data for each trial was performed using the DepthAnalysis function within the DataAnalyzer module of the CichlidBowerTracker.py script. Depth analysis included the following: (i) conversion of raw depth data to “millimeters from Kinect”, (ii) smoothing depth data by applying a Savitsky–Golay filter to spatial and temporal dimensions of raw depth data using the savgol function in Python, (iii) frame-to-frame subtraction (and visualization) of smoothed data at whole trial, daily, and hourly timescales, (iv) identification of above-threshold depth change (whole trial: ± 1.0 cm, daily: ± 0.5 cm, hourly: ± 0.18 cm) regions at each of these timescales, (v) identification of the single highest change region (bower ROI) at each of these timescales, and (vi) calculation of several indices of structural change at these timescales: pixel size of above-threshold depressed (pit-like) and elevated (castle-like) regions; volume of above-threshold depressed (pit-like) and elevated (castle-like) regions; and four calculations of the “Bower Index” (the net volume change divided by the absolute volume change): the overall Bower Index for all depth change, and three for above-threshold change only using sequentially increasing depth thresholds (Trial: 1.0 cm, 3.0 cm, 5.0 cm; Day: 0.4 cm, 0.8 cm, 1.2 cm; 2-h: 0.2, 0.4, 0.8). The final Bower Index used for analyses was the average of these four calculations.

#### Video analysis

Sand disturbance events were identified from video data as described previously^24^. Briefly, this pipeline uses (i) a Hidden Markov Model (HMM) algorithm to detect changes in pixel values through time, and (ii) a density-based clustering algorithm to identify clusters of HMM+ pixels, or putative sand change events. The pipeline thus identifies spatiotemporal clusters of HMM+ pixels, representing putative sand change events. Small video clips centered spatially and temporally around each putative sand change event were classified into different behavioral categories by a trained 18-layer 3D ResNet designed for action recognition as described previously^24^.

### Machine learning

#### Identification of fish from video frames using deep learning

Although behaviors were accurately classified from video data without tracking fish, we were also interested in identifying and counting fish in video data to investigate the social contexts in which these behaviors are expressed. To do this, we manually annotated ~ 1800 frames sampled randomly from videos spanning the same seven trials used for behavior classification, and we trained and tested a Faster region-based convolutional neural network (Faster-RCNN) to identify and count fish. Briefly, Faster-RCNNs involve a two-step neural network approach whereby regions of interest (ROIs) in image data are identified in the first step, and then the ROIs are classified as the target object (in this case, individual fish), or not, in a second step. We used 80% of annotated frames (1473 images) as training data, and 20% (369 images) as test data. Following training, the model performed with a mean average precision for object detection of 0.9935 and an accuracy of 95.47% on the test data.

### Statistics

All statistics were performed using Python 3 (version 3.6 or later) and R (version 3.4.4 or later).

#### Depth change by condition

Sand displacement between conditions was compared using whole trial depth change as the outcome variable and condition (empty tank trials vs. “no bower” control trials vs. bower trials) as the predictor variable. We tested the assumption of heterogeneity of variance using the Fligner–Killeen test, which revealed unequal variance among groups. Based on this, we tested differences between groups using the Kruskal–Wallis H test (non-parametric one-way ANOVA on ranks). Post-hoc pairwise Wilcoxon Rank Sum Tests were performed to assess pairwise significance among groups.

#### Depth change thresholds

To identify depth change thresholds, we quantified whole trial, daily, and hourly depth change across a large and representative sample of control (n = 22) and bower (n = 27) trials. To filter out signals due to noise, we set a minimum size threshold of 1000 contiguous pixels (~ 10 cm^3^) using remove_small_objects within the morphology module in the scikit-image library for Python (for example usage see DepthProcessor.py code at https://github.com/ptmcgrat/Kinect2/blob/master/Modules/Analysis/DepthProcessor.py). We then incrementally applied depth change thresholds in 0.1 mm steps to identify the maximum values that could be expected in the absence of bower construction. These thresholds turned out to be 1.8 mm for hourly change, 5.0 mm for daily change, and 10.0 mm for whole trial change.

#### Bower Index by species

The Bower Index was calculated as the sum of above threshold depth change (directional; positive and negative changes cancel out) divided by the sum of total depth change (absolute value; change in either direction is considered positive) at each timescale. To account for variation in building intensity between individuals, we applied stepped increases in the depth threshold at each timescale, and we averaged together the bower indices calculated using each threshold. Bower indices were compared between species (MC vs. CV vs. TI) using one-way ANOVA and significance of pairwise comparisons were analyzed with post-hoc Tukey’s HSD tests.

#### Spatial covariance of CNN-predicted events and depth change

To quantify the spatial relationship between depth change and behavioral events, we performed correlation analysis on depth change data as well as CNN-predicted behavioral data from seven trials (n = 556,017 total behavioral events). For each trial, we calculated kernel density estimates of the spatial distribution for each of the ten CNN-predicted behavioral categories using the KernelDensity class from the scikit-learn python library, as well as the absolute value of the total depth change at each pixel. We examined the underlying HMM clusters to determine the approximate location (center) and extend (radius) of the associated sand change event and mapped these values into the depth data coordinate system using an affine transformation matrix. We then fitted a 2D kernel density estimation (KDE) to this collection of center-points, using a Gaussian kernel with a bandwidth equal to half of the mean estimated radii. This bandwidth selection process ensured that each KDE would reflect differences in the size of sand-change events unique to individual fish and different behaviors. Next, the KDE and absolute depth-change matrices were concatenated, pixels associated with missing data were excluded, and pairwise correlation between the ten behaviors and depth change volume were calculated. Correlation values were organized into a heatmap using Seaborn’s clustermap function with default settings (hierarchical clustering using a Euclidean distance metric and the UPGMA clustering algorithm). In addition, we calculated the associated p-values using the pearsonr function from the scipy library.

#### Temporal covariance of CNN-predicted events and depth change

To quantify patterns of temporal covariance among different behavioral categories and total depth change, we performed correlation analyses on depth change data as well as events that were previously classified across seven behavioral trials described above under “Spatial covariance of CNN-predicted events and depth change” section. Each of the seven trials was first divided into 60-min time bins. Within each 60-min bin, the number of events was calculated for each category, as well as the total absolute volume of depth change (cm^3^). Bins in which there were zero observations of any behavior were excluded from analysis to prevent inflated correlation values driven by lights-off periods. Pairwise behavior-behavior and behavior-depth change correlations were then performed across all bins and trials (pooled), and Pearson’s R values and corresponding p-values were calculated. Heatmaps were generated using the clustermap function in the Seaborn package for Python, with default settings.

#### Fish counts across behavioral contexts

To analyze differences in number of fish present in frames associated with different behavioral contexts, we used a linear mixed-effects model with fish count as the outcome variable, behavior as a fixed effect, and day nested within subject nested within species as a random effect. Thus the model was as follows:

$${\mathbf{count}}\sim {\text{ behavior }} + \, \left( {{\text{species}}/{\text{subject}}/{\text{day}}} \right).$$

## Supplementary information

Supplementary Information 1.

Supplementary Information 2.

Supplementary Information 3.

Supplementary Tables.

## References

[1] Ache JM (2019). Neural basis for looming size and velocity encoding in the Drosophila giant fiber escape pathway. Curr. Biol..

[2] Rosenthal SB, Twomey CR, Hartnett AT, Wu HS, Couzin ID (2015). Revealing the hidden networks of interaction in mobile animal groups allows prediction of complex behavioral contagion. Proc. Natl. Acad. Sci..

[3] Milner-Gulland E, Fryxell JM, Sinclair AR (2011). Animal Migration: A Synthesis.

[4] Mouritsen H (2018). Long-distance navigation and magnetoreception in migratory animals. Nature.

[5] Russell AL, Morrison SJ, Moschonas EH, Papaj DR (2017). Patterns of pollen and nectar foraging specialization by bumblebees over multiple timescales using RFID. Sci. Rep..

[6] Genzel D, Yovel Y, Yartsev MM (2018). Neuroethology of bat navigation. Curr. Biol..

[7] Prat Y, Yovel Y (2020). Decision making in foraging bats. Curr. Opin. Neurobiol..

[8] Robinson GE, Fernald RD, Clayton DF (2008). Genes and social behavior. Science.

[9] Ben-Shahar Y, Robichon A, Sokolowski MB, Robinson GE (2002). Influence of gene action across different time scales on behavior. Science.

[10] Feng NY, Fergus DJ, Bass AH (2015). Neural transcriptome reveals molecular mechanisms for temporal control of vocalization across multiple timescales. BMC Genomics.

[11] Egert-Berg K (2018). Resource ephemerality drives social foraging in bats. Curr. Biol..

[12] Barber I, Nairn D, Huntingford FA (2001). Nests as ornaments: revealing construction by male sticklebacks. Behav. Ecol..

[13] Odling-Smee FJ, Laland KN, Feldman MW (1996). Niche construction. Am. Nat..

[14] Kocher TD (2004). Adaptive evolution and explosive speciation: the cichlid fish model. Nat. Rev. Genet..

[15] Johnson ZV (2019). Microhabitat predicts species differences in exploratory behavior in Lake Malawi cichlids. bioRxiv.

[16] Maan ME, Sefc KM (2017). Seminars in Cell & Developmental Biology.

[17] Hulsey C, Mims M, Parnell N, Streelman J (2010). Comparative rates of lower jaw diversification in cichlid adaptive radiations. J. Evol. Biol..

[18] Baran NM, Streelman JT (2020). Ecotype differences in aggression, neural activity and behaviorally relevant gene expression in cichlid fish. Genes Brain Behav..

[19] York RA (2015). Evolution of bower building in Lake Malawi cichlid fish: phylogeny, morphology, and behavior. Front. Ecol. Evol..

[20] McKaye KR, Stauffer JR, Turner GF, Konings A, Sato T (2001). Fishes, as well as birds, build bowers. J. Aquaricult. Aquat. Sci..

[21] Konings A (2007). Lake Malawi Cichlids in Their Natural Habitat.

[22] York RA (2018). Behavior-dependent cis regulation reveals genes and pathways associated with bower building in cichlid fishes. Proc. Natl. Acad. Sci. U.S.A..

[23] Mankoff KD, Russo TA (2013). The Kinect: a low-cost, high-resolution, short-range 3D camera. Earth Surf. Proc. Land..

[24] Long L (2020). Automatic classification of cichlid behaviors using 3D convolutional residual networks. iScience.

[25] Qiu, Z., Yao, T. & Mei, T. in *proceedings of the IEEE International Conference on Computer Vision*, 5533–5541.

[26] Hansell M (2000). Bird Nests and Construction Behaviour.

[27] James NL (1969). Nest-building behavior in three species of deer mice, peromyscus. Behaviour.

[28] Borgia G, Pruett-Jones SG, Pruett-Jones MA (1985). The evolution of bower-building and the assessment of male quality. Z. für Tierpsychol..

[29] Hughey LF, Hein AM, Strandburg-Peshkin A, Jensen FH (2018). Challenges and solutions for studying collective animal behaviour in the wild. Philos. Trans. R. Soc. B Biol. Sci..

[30] Robie AA, Seagraves KM, Egnor SR, Branson K (2017). Machine vision methods for analyzing social interactions. J. Exp. Biol..

[31] Dell AI (2014). Automated image-based tracking and its application in ecology. Trends Ecol. Evol..

[32] Macfarlane NBW, Howland JC, Jensen FH, Tyack PL (2015). A 3D stereo camera system for precisely positioning animals in space and time. Behav. Ecol. Sociobiol..

[33] Ardekani R (2013). Three-dimensional tracking and behaviour monitoring of multiple fruit flies. J. R. Soc. Interface.

[34] Weissbrod A (2013). Automated long-term tracking and social behavioural phenotyping of animal colonies within a semi-natural environment. Nat. Commun..

[35] Wiltschko AB (2015). Mapping sub-second structure in mouse behavior. Neuron.

[36] Hong W (2015). Automated measurement of mouse social behaviors using depth sensing, video tracking, and machine learning. Proc. Natl. Acad. Sci. U.S.A..

[37] DiRienzo N, Dornhaus A (2017). Temnothorax rugatulus ant colonies consistently vary in nest structure across time and context. PLoS ONE.

[38] Genise JF, Genise JF (2017). Basic architecture of soil nesting wasps and bees. Ichnoentomology: Insect Traces in Soils and Paleosols.

[39] Theraulaz G, Bonabeau E, Deneubourg JL (1998). The origin of nest complexity in social insects. Complexity.

[40] Metz HC, Bedford NL, Pan YL, Hoekstra HE (2017). Evolution and genetics of precocious burrowing behavior in *Peromyscus* mice. Curr. Biol..

[41] Khuong A (2016). Stigmergic construction and topochemical information shape ant nest architecture. Proc. Natl. Acad. Sci..

[42] Field KE, Maruska KP (2017). Context-dependent chemosensory signaling, aggression and neural activation patterns in gravid female African cichlid fish. J. Exp. Biol..

[43] Wood RI, Newman SW (1995). Integration of chemosensory and hormonal cues is essential for mating in the male Syrian hamster. J. Neurosci..

[44] Anderson DJ, Perona P (2014). Toward a science of computational ethology. Neuron.

