# Supplementary material for: Automated measurement of long-term bower behaviors in Lake Malawi cichlids using depth sensing and action recognition
Source: Sci Rep. 2020 Nov 25;10:20573. doi: 10.1038/s41598-020-77549-2 (PMC7688978; doi:10.1038/s41598-020-77549-2)
Supplement: Supplementary file 1 — Supplementary Information 1. [file 41598_2020_77549_MOESM1_ESM.docx]

**Automated measurement of long-term bower behaviors in Lake Malawi cichlids using depth sensing and action recognition**

Zachary V Johnson^1^, Manu Tej Sharma Arrojwala^1^, Vineeth Aljapur^1^, Tyrone Lee^1^, Tucker J. Lancaster^1,2^, Mark C Lowder^1^, Karen Gu^1^, Joseph Stockert^1^, Rachel L Lecesne^3^, Jean Moorman^3^, Jeffrey T Streelman^# 1,2^, and Patrick T McGrath^# 1,2,4,5^

^1^School of Biological Sciences, Georgia Institute of Technology, Atlanta, GA 30332, USA

^2^Interdisciplinary Graduate Program in Quantitative Biosciences, Georgia Institute of Technology, Atlanta, GA 30332, USA

^3^Parker H. Petit Institute of Bioengineering and Bioscience, Georgia Institute of Technology, Atlanta, GA 30332, USA

^4^Department of Computer Science, Georgia Institute of Technology, Atlanta, GA 30332, USA

^5^School of Physics, Georgia Institute of Technology, Atlanta, GA 30332, USA

^#^Co-corresponding authors: [patrick.mcgrath@biology.gatech.edu](mailto:patrick.mcgrath@biology.gatech.edu) (P.T.M.), [todd.streelman@biology.gatech.edu](mailto:todd.streelman@biology.gatech.edu) (J.T.S.)

**Supplementary Methods and Materials**

**System Design**

Animal care guidelines required that testing over such extended time periods had to be done in the home tank (as opposed to external testing arenas). In our facilities, home tanks are supported on tank racks with built-in piping and support beams that partially occlude top-down fields of view (FOVs) (e.g. see Supplementary Figure 1). Additionally, all tanks have a central support crossbeam that partially occludes top-down FOVs. We found that a ~36 cm diameter sand tray placed on one half of the home tank provided a sufficient volume of sand for males to construct bowers, and was small enough to fit into an unobstructed top-down FOV (Supplementary Figure 3B). We designed a custom acrylic platform to surround the sand tray to prevent subjects from spitting sand over the edge of the tray onto the bottom of the aquarium. Thus, in this design subject males and females could freely enter and exit the sand tray region throughout the trial.


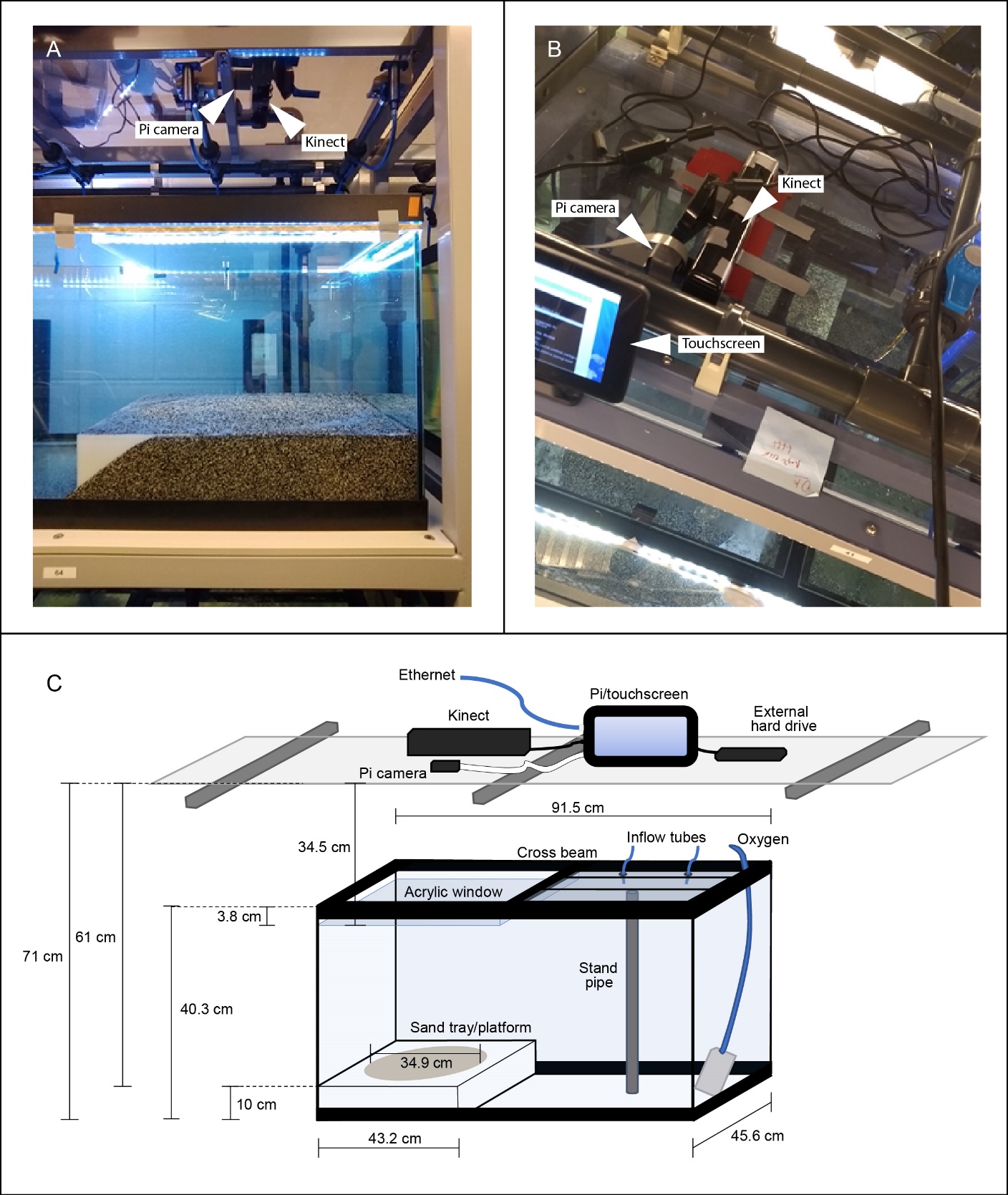


**Supplementary Figure 1. Photographs, schematic, and measurements of behavioral paradigm.** Photographs (A-B) and detailed dimensions of home tank setup for bower behavior assays (C). The final design had to be compatible with several pre-existing physical constraints such as tank rack support beams (gray metal beams visible just beneath acrylic in A, B), water inflow lines (gray acrylic and blue rubber tubes above and below transparent acrylic top, visible in A and B), and aquarium cross beams (black plastic cross beam visible in B). All electronic equipment was placed on top of a transparent acrylic shelf above the tank rack, with the Kinect and Raspberry Pi camera (indicated with white arrows in A, B) aimed downwards for a top-down view of the sand tray.

**Controller Spreadsheet**

To avoid the need for manual control of recording equipment above behavior tanks, we created custom software to remotely control each unit using a single Google Spreadsheet: each Raspberry Pi monitored one of the rows of the spreadsheet for commands (Record, Rewrite, Stop, etc.) and executed accordingly (Supplementary Figure 3). The Pi also continuously forwarded analyses of depth change over the previous hour, day, and whole trial to the Google spreadsheet for remote visualization of bower activity (Supplementary Figure 4). This system thus allows for real time monitoring of bower construction.

To setup the Google spreadsheet, two different Python APIs were used to easily access Google APIs: Gspread and PyDrive. Gspread is a module that specifically manages Google Spreadsheets, while Pydrive manages files more generally in Google Drive. In our setup, PyDrive was used to upload .jpeg files containing snapshots and summary images to Google Drive, and Gspread was used to read and write directly to the Controller sheet. The latest documentation and downloads for Gspread are available here: (<https://gspread.readthedocs.io/en/latest/index.html>) and for Pydrive here: ([https://pythonhosted.org/PyDrive/#](https://pythonhosted.org/PyDrive/)).

A new Google account was created to house the Google Spreadsheet. We recommend for several reasons. First, this limits the possible exposure of a personal Gmail account since different authentication keys or tokens will need to be distributed to each system that requires access. Second, a new account may also be useful if an automated email system is implemented because it can act as the originating email address that all Pi systems can access.

All authentication for Google APIs goes through OAuth2, but these two modules require different credentials. Gspread requires a Service Account Key, and Pydrive requires a client secret .json file. The latest instructions on how to obtain these credentials and how to use them for authentication can be found in these modules’ documentations, for (Gspread: <https://gspread.readthedocs.io/en/latest/oauth2.html>, and for Pydrive: <https://gsuitedevs.github.io/PyDrive/docs/build/html/quickstart.html#authentication>.

After obtaining the appropriate credentials, each Pi needs to have both Gspread and Pydrive downloaded and installed, the service account key for Gspread, the client secret .json file for Pydrive, and an internet connection. This basic setup can easily be customized to fit other experiments in several ways that include but not are limited to adding or changing the modules used and changing the organization and information relayed to the Controller Spreadsheet.

**Automated Email System**

An automated email system was setup to send summary updates of the current status for all Pi systems at the beginning and end of each day, as well as real-time notifications of when recordings were unexpectedly interrupted. The basic procedure of this python script is to first check the Controller sheet for nonresponsive Pi systems or to check on the status of all the Pi systems for a summary update. The information from this check is stored and then written into an email which is sent through the Google account’s Gmail. To run this procedure, the Python script was run continuously on a single Pi system with internet connection, the Service Account Key for Gspread, and a .txt file containing the username, password, and email addresses of recipients. The essential modules for the script were Gspread for reading into the Controller sheet and smtplib for sending the email. More information about smtplib and an example of how to use this module can be found here: <https://docs.python.org/3/library/smtplib.html>.

_­_



**Supplementary Figure 2. Google Drive Controller spreadsheet for remote control of Raspberry Pi systems.** All Raspberry Pi systems were remotely controlled through a Google Drive Spreadsheet. The master spreadsheet comprised multiple sub-sheets for organizing trial information. The first sheet, “RaspberryPi” shown above, was used to remotely issue commands to each Pi unit through a Command Column including Start, Stop, Restart, Rewrite, Upload (to Dropbox), Delete, and Snapshots (shown in blue outlined box above). The current status of each Pi was continuously updated in a separate “Status” column (all green cells reading “Running” indicate actively recording trials). An “Error” column displayed errors encountered during interruptions to help with troubleshooting and debugging. The “Ping” column registered pings from each Pi released every five minutes, and could also be used to identify interruptions. The final “Image” column updates every five minutes provides RGB and depth snapshots to enable live monitoring of depth change across the whole trial, the previous day, and the previous hour. A higher resolution version of this figure is included in supplemental.


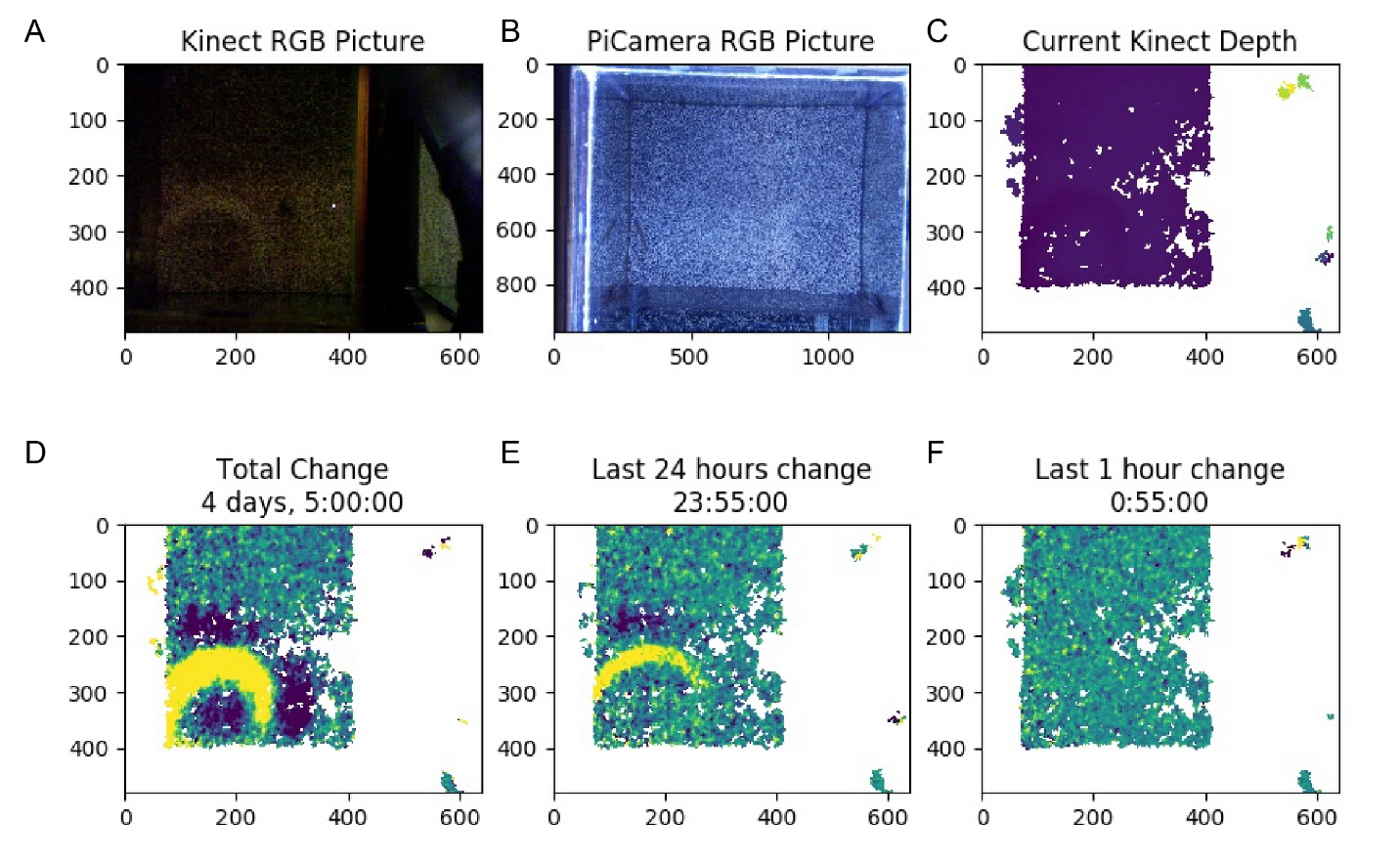


**Supplementary Figure 3. Example screenshot of live update of depth change in behavior tank.** Full view of .jpeg file generated every five minutes in the Image column of the Google Controller spreadsheet. The file contains an RGB image captured by the Kinect (A), and RGB image captured by the Raspberry Pi camera (B), the current depth across the sand surface (C), the total depth change across the whole trial as well as the current duration of the trial (D), depth change in the previous 24 hours (E), and depth change in the previous hour (F). Labels on x- and y-axes indicate pixel dimensions.

Depth Sensing System Validation

*The Kinect measures the distance of the sand tray surface through water*

The Kinect depth sensor records both depth data and RGB data across the FOV. This sensor was designed for detecting depth changes through air (i.e. Microsoft Xbox users playing video games in their living rooms). In preliminary experiments, we tested how the ~27 cm of water between the Kinect and the sand tray would interfere with its ability to measure distances of surfaces along the bottom of aquarium tanks. We found that individual snapshots of the sand surface contained a large amount of missing data, potentially due to reflection at the water surface boundary and absorption by water. For example, in a sample set of raw snapshot frames, we found that 40.0 ± 0.04% of pixels per frame contained missing data (Fig. 2A). To improve our measurements of the sand surface, we modified our protocol to collect five minutes worth of snapshots in rapid succession (~10 fps) and average them into a single frame. Although this reduced the temporal resolution of depth sensing, this limitation was reasonable because we expected structural changes of interest to occur over the course of hours. Averaging drastically reduced the number of NaN pixels in each frame (Fig. 2B; proportion of NaN values decreased to 20.6 ± 0.06%). We also applied spatial interpolation (see Methods) to estimate values in small regions of missing data, which further reduced the proportion of NaN pixels to 10.8% for the final analyzed dataset (Fig. 2C). Thus, our pipeline generated depth data across ~90% of the sand tray surface every five minutes, enabling analysis of surface change through time (Fig. 2D-E).

*Thresholds improve signal-to-noise for measuring bower construction*

We detected significant depth change signals in empty tanks and in control trials, presumably due to noise and other behaviors that alter the sand surface, respectively. Based on these results, we tested if thresholds could separate signals caused by bower construction from signals caused by noise and other non-bower behaviors. We measured the maximum whole trial volume change signals observed in empty tank and control trials (this turned out to be 1.0 cm), and then tested whether volume change signals in bower trials exceeded this threshold. Indeed, we identified greater depth change signals in every bower trial (29/29; Fig. 2F), suggesting that threshold could be used to filter out low magnitude depth change signals caused by noise and other non-bower behaviors (Fig. 2G).

*Measurement of bower activity on shorter timescales*

We next tested whether bouts of bower activity within trials could be detected on shorter timescales by analyzing depth change over 24-hour and 2-hour periods. We used a similar approach to identify thresholds that separated depth change during bower trials from depth change during control trials. Again, we found thresholds that separated daily and hourly depth change in bower trials versus control trials. Overall, 160/264 (60.6%) of all days analyzed, and 538/3,168 (17.0%) of all 2-hour bins analyzed contained depth change exceeding these thresholds (Fig. 5A). Frame-to-frame subtraction of depth data in 5-minute intervals further revealed sharp peaks in activity punctuated throughout whole trials (e.g. see Fig. 5B, representative castle-building MC trial).


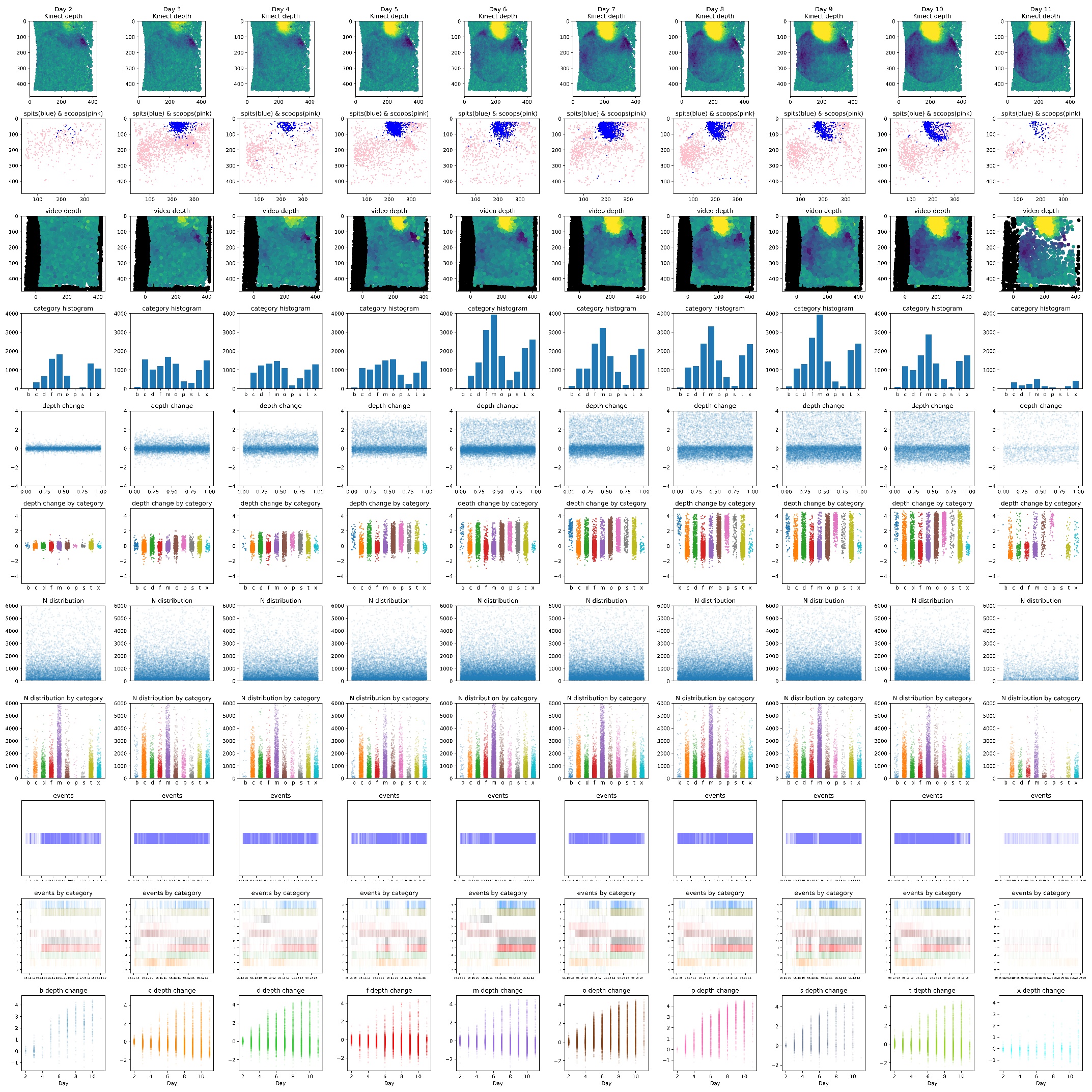


**Supplementary Figure 4. Example output following analysis of registered 3D ResNet-predicted behavioral events with depth sensing data across full trials.** Visualization of behavioral analyses of a representative *Mchenga conophoros* trial. Depth change by day as measured by the Kinect across the full trial (first row). Spatial location of all 3D ResNet-predicted bower scoop (pink) and bower spit (blue) events across the full trial (second row). Depth of all behavioral events by day across the full trial (third row). Number of events across categories by day (fourth row). Depth change at locations of all behavioral events by day (fifth row). Depth change at locations of all behavioral events by category across days (sixth row). Pixel size of all sand change clusters by day (seventh row). Pixel size of all behavioral events by category across days (eighth row). Temporal distribution of all behavioral events by day (ninth row). Temporal distribution of all behavioral events by category across days (tenth row). Sand surface height at location of each behavioral event across days (consecutive data columns within each plot), by category (each consecutive plot represents a different behavioral category; eleventh row). Letter abbreviations for behaviors are as follows: b = bower multiple, c = bower scoop, d = sand dropping, f = feed scoop, m = feed multiple, o = fin swipe/other, p = bower spit, s = quiver/spawn, t = feed spit, x = no fish. A higher resolution of this figure is included in supplemental.

**Supplementary Table S1. Pairwise correlations between behavior categories and depth change through time.** Behavioral categories are indicated in columns “x” and “y”. The Pearson’s R correlation value is indicated in column “R”. The p-value associated with the correlation is indicated in column “p”. Abbreviations for behavioral categories are as follows: c = bower scoop, p = bower spit, b = bower multiple event, f = feed scoop, t = feed spit, m = feed multiple event, s = quiver/spawn, d = sand dropping, o = fin swipe/other, x = no fish, depth = whole trial absolute volume change.

**Supplementary Table S2. Pairwise spatial correlations between behavior categories and depth change.** Behavioral categories are indicated in columns “x” and “y”. The Pearson’s R correlation value is indicated in column “R”. The p-value associated with the correlation is indicated in column “p”. Abbreviations for behavioral categories are as follows: c = bower scoop, p = bower spit, b = bower multiple event, f = feed scoop, t = feed spit, m = feed multiple event, s = quiver/spawn, d = sand dropping, o = fin swipe/other, x = no fish, depth = whole trial absolute volume change.
