# Supplementary material for: Automated measurement of long-term bower behaviors in Lake Malawi cichlids using depth sensing and action recognition
Source: Sci Rep. 2020 Nov 25;10:20573. doi: 10.1038/s41598-020-77549-2 (PMC7688978; doi:10.1038/s41598-020-77549-2)
Supplement: Supplementary file 4 — Supplementary Tables. [file 41598_2020_77549_MOESM4_ESM.docx]

| Species | Condition | n | Bower shape | Description |  |
| --- | --- | --- | --- | --- | --- |
| *-* | Empty | 9 | - | No fish in tank |  |
| *CV* | Feeding only | 3 | - | Four female fish |  |
| *MC* | Feeding only | 3 | - |  |  |
| *TI* | Feeding only | 3 | - |  |  |
| *CV* | Bower | 9 | Pit | One male and four female fish |  |
| *MC* | Bower | 7 | Castle |  |  |
| *TI* | Bower | 5 | Pit |  |  |
| *MC/CV F_1_* | Bower | 3 | Pit/Castle |  |  |
| *TI/MC F_1_* | Bower | 5 | Pit/Castle |  |  |

**Supplementary Table 3.** Summary of behavioral trials analyzed for validation of depth sensing system. Abbreviations: CV = *Copachromis virganialis*; MC = *Mchenga conophoros*; TI = *Tramitichromis intermedius*. F_1_ hybrids display codominant phenotype. Males initially build a pit structure and then transition to build a castle structure nearby the original pit.

| Species | Condition | n | Bower shape | Description |  |
| --- | --- | --- | --- | --- | --- |
| *CV* | Bower | 1 | Pit | One male and four female fish |  |
| *MC* | Bower | 3 | Castle |  |  |
| *TI* | Bower | 2 | Pit |  |  |
| *MC/CV F_1_* | Bower | 2 | Pit/Castle |  |  |

**Supplementary Table 4.** Summary of behavioral trials used for analysis of registered video and depth data. Abbreviations are the same as in Supplementary Table 3.
